# Remarks on the Cure of Spasmodic Asthma

**Published:** 1799-10

**Authors:** 


### *Remarks on the Cure of Spafmodic Afthma.*

[Concluded from our last Number, pp. 169, 170.]

In fpafmodic afthma, arifing from tubercles of the lungs, a large blifter applied to the fhoulders gives eafe to the breaft, and promotes expectoration. After the paroxyfm, emetics are ufeful; fometimes a purgative, confifting of manna, Glauber's falt, and foluble tartar, has fpeedily terminated the fit. A more immediate, though only temporary relief, may be given to the patient by fpirit of hartfhorn, effence of caftoreum, or a folution of afa fœtida in pennyroyal-water, of which, a table-fpoonful muft be given every five hours.

A more effectual method, however, ought to be adopted, in order to refolve gradually the tubercles of the lungs, and the vifcid mucus fecreted by the glands, which fo frequently occafions afthmatic fymptoms, and chronic dyfpnœa. If the ufual refolvent falts fhould not produce the defired effect, a folution of the *muriat of barytes* muft be adminiftered in a proper vehicle. Indeed, pure water mixed with the eighth part of vinegar, and fweetened with with honey orfugar, has alfo proved beneficial in a paroxyfm of afthma, if drank in fufficient quantity; but for patients with whom acids do not agree, neither this acidulated water, nor the oxymel of fquills are proper, but the volatile alkali and afa fœtida are generally efficacious.

With a view to prevent the return of fpafmodic afthma, a great variety of .emedies have been prefcribed with fuccefs, according to the different conftitutions and circumftances of patients: the principal of thefe are, the bark, the chalybeates, vitriolic elixir, country air, equitation, a flannel waiftcoat worn next the fkin, fetons, and the long continued ufe of the common pilulæ fcillæ.

